# Neonatal mortality in Ethiopia: a protocol for systematic review and meta-analysis

**DOI:** 10.1186/s13643-019-1012-x

**Published:** 2019-04-26

**Authors:** Tesfalidet Tekelab, Mohammed Akibu, Negash Tagesse, Temesgen Tilhaun, Yosef Yohanes, Smriti Nepal

**Affiliations:** 1 Institute of Health sciences, Wollega University, Nekemte, Ethiopia; 2 School of Medicine and Public Health, University of Newcastle, Newcastle, Australia; 3 Department of Midwifery, Institute of Medicine and Health Sciences, Debre Berhan University, Debre Berhan, Ethiopia; 4 Department of Paediatrics, Hawassa University, Hawassa, Ethiopia; 5 Hawassa College of Health Science, Hawassa, Ethiopia

**Keywords:** Neonatal mortality, Causes of neonatal mortality, Meta-analysis, Ethiopia

## Abstract

**Background:**

A child’s risk of dying is highest in the neonatal period, i.e. the first 28 days of life. Newborn death accounts for nearly half of under-five death. More than 80% of newborn deaths are the result of preventable and treatable conditions. Ethiopia has made significant progress towards reducing under-five mortality; however, the rate of neonatal mortality (NMR) still accounts for 41% of under-five deaths. With this systematic review and meta-analysis, we aim to determine the magnitude, causes, and determinants of neonatal mortality in Ethiopia.

**Methods:**

We will conduct a comprehensive search of the following electronic databases: PubMed, MEDLINE, EMBASE, CINAHL, Google Scholar, and maternity and infant care databases as well as grey literature. We will assess the quality of studies by using Newcastle-Ottawa Scale (NOS) checklist. Two reviewers will screen all retrieved articles, conduct data extraction, and then critically appraise all identified studies. We will analyse data by using STATA 11 statistical software. We will demonstrate pooled estimates and determinants of neonatal mortality with effect size and 95% confidence interval.

**Discussion:**

The result from this systematic review will inform and guide health policy planners and researchers on the burden, causes, and determinants of neonatal mortality in Ethiopia. To our knowledge, this is the first systematic review in Ethiopia. We will synthesise the findings to generate up-to-date knowledge on neonatal mortality in Ethiopia.

**Systematic review registration:**

PROSPERO-CRD42018099663

**Electronic supplementary material:**

The online version of this article (10.1186/s13643-019-1012-x) contains supplementary material, which is available to authorized users.

## Background

Child survival should remain at the heart of global health and development goals [1]. Globally, 2.6 million newborns die within the first month of life, annually. Newborn death accounts for nearly half of under-five deaths [2]. Even though there is a global decrease in neonatal mortality, the decrease is slower in neonatal mortality rates (NMRs) compared to under-five mortality rates [1, 2]. Approximately 7000 newborns die daily; most of these deaths occur within the first week with one million taking their first and last breaths on the day they are born and one million dying within the next 6 days [3, 4]. Most newborn deaths (99%) occur in low-income countries with half occurring at home [5]. Sub-Saharan Africa and South Asia each account for 39% of all global neonatal deaths [3, 6]. In sub-Saharan Africa, the NMR is 31 per 1000 live births whereas the global NMR is 20 per 1000 live births [7]. Newborn deaths can be prevented through effective strategies such as skin-to-skin contact, early breast feeding initiation, newborn resuscitation, kangaroo mother care for premature babies, clean water, disinfectant, and good nutrition along with access to well-trained healthcare providers [8]. More than 80% of newborn deaths are the result of premature birth complication and infections such as sepsis, meningitis, and pneumonia [3, 4, 9].

Ethiopia has made remarkable progress in achieving many of the national health indicators. The implementation of the National Child Survival Strategy (2005–2015) helped in the reduction of child mortality; however, the current under-five and neonatal mortality rates remain unacceptably high [10]. Between 2000 and 2016, under-five mortality in Ethiopia decreased from 166 to 67 per 1000 live births (reduction of 60%). However, NMR is decreasing at a slower rate and now accounts for 41% of under-five deaths [11–13]. The leading causes for neonatal death in Ethiopia are prematurity, asphyxia, and neonatal sepsis [10, 14, 15].

Researchers have stated that neonatal mortality rate to be a standard indicator for evaluation of health status of a country [16, 17]. Therefore, it is important to explore the factors that contribute to neonatal death. Moreover, identifying the cause of death and cause-specific contributions to neonatal mortality is important in selecting strategies to further reduce newborn deaths. Studies have shown that neonatal mortality is influenced by several factors [15, 18]. None of the studies have shown pooled estimates of neonatal mortality and its determinants in Ethiopia. Thus, there is a strong need to systematically evaluate the existing evidence on determinants of neonatal mortality on which one can base health policy planning.

### Objectives and research questions

The objectives of this study are (1) to show the pooled prevalence of neonatal mortality in Ethiopia and (2) to synthesise evidence on the determinants and causes of neonatal mortality in Ethiopia.

This systematic review and meta-analysis is guided by the following research questions:

(1) What is the pooled estimate of neonatal mortality in Ethiopia? (2) What are the determinants and causes of neonatal mortality in Ethiopia?

## Methods

### Reporting of the review findings

We developed this protocol in accordance with the Preferred Reporting Items for Systematic review and Meta-analysis Protocols (PRISMA-P) statement [19] (Additional file 1). The protocol for this review was registered on PROSPERO (“CRD42018099663”) on 19 September 2018. We will use the Preferred Reporting Items for Systematic review and Meta-analyses (PRISMA-2009) statement to report the findings [20].

### Inclusion criteria

We will include cross-sectional, case-control, and cohort studies that have reported on neonatal mortality and have been published in peer-reviewed journals. In addition, we will include findings from regional or national survey reports. We will include studies that have been conducted in Ethiopia. Our focus will be on studies reporting factors, causes, and the rates of neonatal mortality.

### Exclusion criteria

We will exclude studies published in languages other than English. We will exclude studies that include stillbirth on neonatal mortality. Additionally, we will exclude case reports and expert opinions.

### PECO search guide

*Population*: Live born neonates

*Exposure*: Predictors/determinants of neonatal mortality. The determinants are characteristics or exposures that increase the likelihood of neonatal mortality. These may be related to residence, maternal age, educational status, and antenatal exposure.

*Comparison*: The reported reference group for each predictor/determinant in each study (e.g. neonatal mortality in mother living in urban area versus mothers living in rural area).

*Outcome*: Neonatal mortality is defined as ‘a death occurring during the first four weeks (28 days) after birth of live baby’ [21]. We will include studies that assess perinatal mortality, if the studies have excluded stillbirth from perinatal mortality.

### Search strategy

We will develop an appropriate and comprehensive search strategy with relevant search terms and pilot test it before the final search. We will search PubMed, MEDLINE, EMBASE, CINAHL, Google Scholar, and Ovid Maternity and Infant Care Databases. We will include articles published from start of indexing until 10 August 2018. We will use Medical Subject Heading (Mesh), keywords, and free text search terms. As the search terms, we will include alternative terms for neonatal mortality, and will combine them using Boolean operators. To ensure the comprehensiveness, we will consult an expert librarian. The search strategy for PubMed is supplemented with this protocol (Additional file 2).

We will utilise snowballing to screen the references of identified articles for potentially relevant studies. Furthermore, we will contact experts, researchers, and relevant organisations for suggestions on other existing relevant studies.

### Selection of studies

Two authors (TT and NT) will review the studies, based on inclusion and exclusion criteria. The review will follow three stages. During the first stage, we will assess the titles of the studies identified from the search. Then abstract screening, abstracts of these selected titles will be included for the final stage of full-text screening. During full-text screening, we will screen the full texts of abstracts selected in the previous stage. In the review, we will only include those studies approved by both authors. The authors will resolve disagreements through discussion or consultation with a third reviewer (SN). We will provide reason for exclusion for all excluded studies. We will prepare a final list of articles for data extraction.

### Data extraction and management

Using the Joanna Briggs Institute (JBI) data extraction form for experimental/observational studies [22], we will extract relevant data. We will pretest the data extraction form on four studies of each type, to ensure that it adequately facilitates the collection of all necessary data required for an effective systematic review and meta-analysis.

Two review authors (TT and NT) will extract the data independently. Discrepancies between data extractors will be discussed to reach consensus. If a consensus cannot be reached, the authors will consult a third reviewer (SN). For each included articles, we will record the first author’s last name, year of publication, the setting where the study was conducted, study design, study period, sample size, the response rate, the population, outcome definition, comparison groups, and the effect estimate.

### Quality assessment

Three authors (TT, NT, and YY) will independently conduct quality assessment of included studies, by using the checklist of the Newcastle-Ottawa Scale (NOS) (Additional file 3) for cohort and case-control studies [23]. We will use the adapted version of NOS for cross-sectional studies. Based on NOS, we will award studies a maximum of four stars within the selection, two stars within comparability, and three stars within outcome categories.

### Data synthesis and analysis

We will perform a narrative description of the study population, the studies included, the risk factors identified, and the cause for mortality as well as the outcome characteristics. We will use tables and figures to summarise the selected studies and results. Using STATA 11 statistical software, we will carry out the data entry and statistical analysis. We will demonstrate the pooled prevalence of neonatal mortality in Ethiopia. To examine the possible risk of publication bias, we will use funnel plots and Egger’s test [24]. A *p* value < 0.10 will be considered indicative of statistically significant publication bias. If there is evidence of publication bias, we will use Duval and Tweedie’s trim-and-fill method [25]. We will assess heterogeneity by using chi-squared test on Cochran’s *Q* statistic with a 5% level of statistical significance [26] and *I*^2^ statistic, assuming that *I*^2^ value of 25%, 50%, and 75% being representative of low, moderate, and high heterogeneity, respectively [27]. We will use fixed effect model if the studies have similar methodology, same population, and study design. If not, then, we will utilise the random effect model. If the heterogeneity is significant (*I*^2^ > 50%), then we will conduct subgroup analyses and meta-regression to investigate sources of heterogeneity. If the meta-analysis is not possible, we will conduct narrative synthesis.

### Subgroup and sensitivity analyses

Based on study design, sample size, regions or state, year of publication, quality of studies, and study settings, we will perform subgroup analysis. To conduct sensitivity analysis, we will assess the stability or robustness of the pooled estimates to outliers and the impact of individual studies.

## Discussion

This review will provide a detailed summary of the evidence on factors and causes of neonatal mortality in Ethiopia. This review will be the first to synthesise available data on neonatal mortality. Findings of the review will fill an evidence gap in understanding the burden, risk factors, and causes of neonatal mortality in Ethiopia. The result from this review will inform health policy planners and researchers up-to-date data on neonatal mortality and provide direction in which factors the policy should focus to reduce neonatal mortality in Ethiopia.

### Dissemination plan

The results of this systematic review and meta-analysis will be published in a peer-reviewed journal and presented at national and international research conferences.

## Additional files

Additional file 1: PRISMA-P (Preferred Reporting Items for Systematic review and Meta-Analysis Protocols) 2015 checklist: recommended items to address in a systematic review protocol. (DOCX 23 kb)

Additional file 2: PubMed search string. (DOCX 12 kb)

Additional file 3: **Table S1.** Methodological quality assessment of cohort studies using Newcastle-Ottawa Scale (NOS). (DOCX 15 kb)

## Abbreviation

JBI: Joanna Briggs Institute
NOS: Newcastle-Ottawa scale
PECO: Population, Exposure, Comparison, and Outcome
