# Supplementary material for: Neonatal mortality in Ethiopia: a protocol for systematic review and meta-analysis
Source: Syst Rev. 2019 Apr 26;8:103. doi: 10.1186/s13643-019-1012-x (PMC6486678; doi:10.1186/s13643-019-1012-x)
Supplement: Supplementary file 2 — PubMed search string. (DOCX 12 kb) [file 13643_2019_1012_MOESM2_ESM.docx]

**Neonatal mortality in Ethiopia: A protocol for systematic review and Meta-analysis**

| **Component** | **Search terms** | **n hits****^¥^** |
| --- | --- | --- |
| #1 | (neonatal mortality[MeSH Terms]) OR perinatal mortality[MeSH Terms]) OR Newborn mortality[MeSH Terms]) OR Neonatal death[MeSH Terms]) OR Newborn death[MeSH Terms]) OR perinatal death[MeSH Terms] | 46689 |
| #2 | (determinants[MeSH Terms]) OR factors[MeSH Terms]) OR risk[MeSH Terms]) OR predictors[MeSH Terms])) OR causes[MeSH Terms] | 1089169 |
| #3 | (Ethiopia[MeSH Terms]) OR Ethio"[MeSH Terms | 10020 |
| #4 | #1 OR #2 OR #3 | 49 |

**PubMed search string**

***^¥^*** *=search date is August 6, 2018 at 11:52PM*
