# Supplementary material for: Neonatal mortality in Ethiopia: a protocol for systematic review and meta-analysis
Source: Syst Rev. 2019 Apr 26;8:103. doi: 10.1186/s13643-019-1012-x (PMC6486678; doi:10.1186/s13643-019-1012-x)
Supplement: Supplementary file 3 — Table S1. Methodological quality assessment of cohort studies using Newcastle-Ottawa Scale (NOS). (DOCX 15 kb) [file 13643_2019_1012_MOESM3_ESM.docx]

**Methodological quality assessment**

Table S1: Methodological quality assessment of cohort studies using Newcastle - Ottawa Scale (NOS)

| First author, publication year | Criteria | | | | | | | | |  |
| --- | --- | --- | --- | --- | --- | --- | --- | --- | --- | --- |
|  | **Selection** | | | | **Comparability** | | **Outcome** | | |  |
|  | **Representativeness of the exposure** | **Selection of the non-exposed** | **Ascertainment of exposure** | **Demonstration that outcome of interest was not present at start of study** | **Study controls for most important factor(s)** | **Study controls for second important factor(s)** | **Assessment of outcome** | **Was follow-up long enough for outcomes to occur** | **Adequacy of follow up of cohorts** | **Total score** |
|  |  |  |  |  |  |  |  |  |  |  |
|  |  |  |  |  |  |  |  |  |  |  |
|  |  |  |  |  |  |  |  |  |  |  |
|  |  |  |  |  |  |  |  |  |  |  |
|  |  |  |  |  |  |  |  |  |  |  |
|  |  |  |  |  |  |  |  |  |  |  |

*Note: each item account 1 point.* Studies with less than seven star-items were considered low quality and those with seven star-items or more were considered high quality.

Table S1: Methodological quality assessment of case-control studies using Newcastle - Ottawa Scale (NOS)

| **First author, publication year** | **Criteria** | | | | | | | | |  |
| --- | --- | --- | --- | --- | --- | --- | --- | --- | --- | --- |
|  | **Selection** | | | | **Comparability** | | **Exposure** | | |  |
|  | **Adequacy of case definition** | **Representativeness of the cases** | **Selection of Controls** | **Definition of Controls** | **Study controls for most important factor(s)** | **Study controls for second important factor(s)** | **Ascertainment of exposure** | **Same method of ascertainment for cases and controls** | **Non-Response rate** | **Total score** |
|  |  |  |  |  |  |  |  |  |  |  |
|  |  |  |  |  |  |  |  |  |  |  |
|  |  |  |  |  |  |  |  |  |  |  |
|  |  |  |  |  |  |  |  |  |  |  |
|  |  |  |  |  |  |  |  |  |  |  |
|  |  |  |  |  |  |  |  |  |  |  |
|  |  |  |  |  |  |  |  |  |  |  |
|  |  |  |  |  |  |  |  |  |  |  |
|  |  |  |  |  |  |  |  |  |  |  |

*Note: each item account 1 point.* Studies with less than seven star-items were considered low quality and those with seven star-items or more were considered high quality.

Table S1 Continued: Methodological quality assessment of cross-sectional studies using Newcastle - Ottawa Scale (NOS)

| **First author, publication year** | Criteria | | | | | | | | |  |
| --- | --- | --- | --- | --- | --- | --- | --- | --- | --- | --- |
|  | **Selection** | | | | **Comparability** | | **Outcome** | | |  |
|  | **Representativeness of the sample** | **Non-respondents** | **Sample size** | **Ascertainment of the exposure (risk factor)** | **The study controls for the most important factor** | **The study control for any additional factor** | **Assessment of the outcome** | **Statistical test** |  | **Total score** |
|  |  |  |  |  |  |  |  |  |  |  |
|  |  |  |  |  |  |  |  |  |  |  |
|  |  |  |  |  |  |  |  |  |  |  |
|  |  |  |  |  |  |  |  |  |  |  |
|  |  |  |  |  |  |  |  |  |  |  |
|  |  |  |  |  |  |  |  |  |  |  |

*Note: each item account 1 point.* Studies with less than seven star-items were considered low quality and those with seven star-items or more were considered high quality.
